# Chromosome-level genome of spider *Pardosa pseudoannulata* and cuticle protein genes in environmental stresses

**DOI:** 10.1038/s41597-024-02966-1

**Published:** 2024-01-24

**Authors:** Na Yu, Jingjing Li, Haibo Bao, Yixi Zhang, Zhiming Yang, Fangfang Li, Jingting Wang, Zewen Liu

**Affiliations:** Key Laboratory of Integrated Management of Crop Diseases and Pests (Ministry of Education), College of Plant Protection, Nanjing Agricultural University, Weigang 1, Nanjing, 210095 China

**Keywords:** Genome, Entomology

## Abstract

Spiders are representative arthropods of adaptive radiation. The high-quality genomes have only been reported in several web weaver spider species, leaving the wandering spiders’ genomic information scarce. The pond wolf spider, *Pardosa pseudoannulata*, is a representative species in the retrolateral titial apophysis (RTA) clade. We present a chromosome-level *P. pseusoannulata* genome assembly of 2.42 Gb in size with a scaffold N50 of 169.99 Mb. Hi-C scaffolding assigns 94.83% of the bases to 15 pseudo-chromosomes. The repeats account for 52.79% of the assembly. The assembly includes 96.2% of the complete arthropod universal single-copy orthologs. Gene annotation predicted 24,530 protein-coding genes with a BUSCO score of 95.8% complete. We identified duplicate clusters of Hox genes and an expanded cuticle protein gene family with 243 genes. The expression patterns of CPR genes change in response to environmental stresses such as coldness and insecticide exposure. The high-quality *P. pseudoannulata* genome provides valuable information for functional and comparative studies in spiders.

## Background & Summary

Spiders are considered one of the most diverse terrestrial predators with more than 50,000 species described^1^. They conquer most area of the planet and are well known for their sophisticated use of silk and venom^2^. Moreover, spiders are important model for evolution and adaptive radiation given the varied environmental conditions of their habitats^3^. Taking advantage of the ever expanding genomic information, researchers are able to untangle different aspects of spider biology, with particular interest on venom, silk, and the phylogeny^4–17^. The increasing numbers of genomes will facilitate the phylogenomic study to unravel the roots of spiders and comparative studies to decipher the morphological and behavioural traits of spiders. However, the high-quality genomes are far from adequacy for systemic studies when the species diversity is concerned. Different from various clades of web-weaver spiders, a number of wandering spiders constitute the retrolateral tibial apophysis (RTA) clade barely building foraging webs. This spider lineage is remarkably diverse with over 25,000 species, including the popular families Salticidae (jumping spiders) and Lyosidae (wolf spiders), and thus plays an important role in spider evolution. However, no high-quality genome has been reported in the RTA clade. We here report the pond wolf spider *Pardosa pseudoannulata* (Fig. 2b) genome assembly at the chromosome-level to fill the gap. *P. pseudoannulata* predates on a broad range of insects including planthoppers and leafhoppers^18^ and represent an important natural enemy in agricultural ecosystem, where they encounter various environmental stresses, such as coldness, drought, and insecticides. Our previous studies have revealed that the cuticle proteins are involved in spiders’ adaptation to stresses^19^. Taking advantage of the genome assembly, we conducted a systemic analysis of the expanded gene family encoding the cuticle proteins with R&R Consensus (CPR) and evaluated their involvement in spiders’ adaptation to coldness during overwintering and to insecticide nitenpyram exposure.

**Fig. 1:**
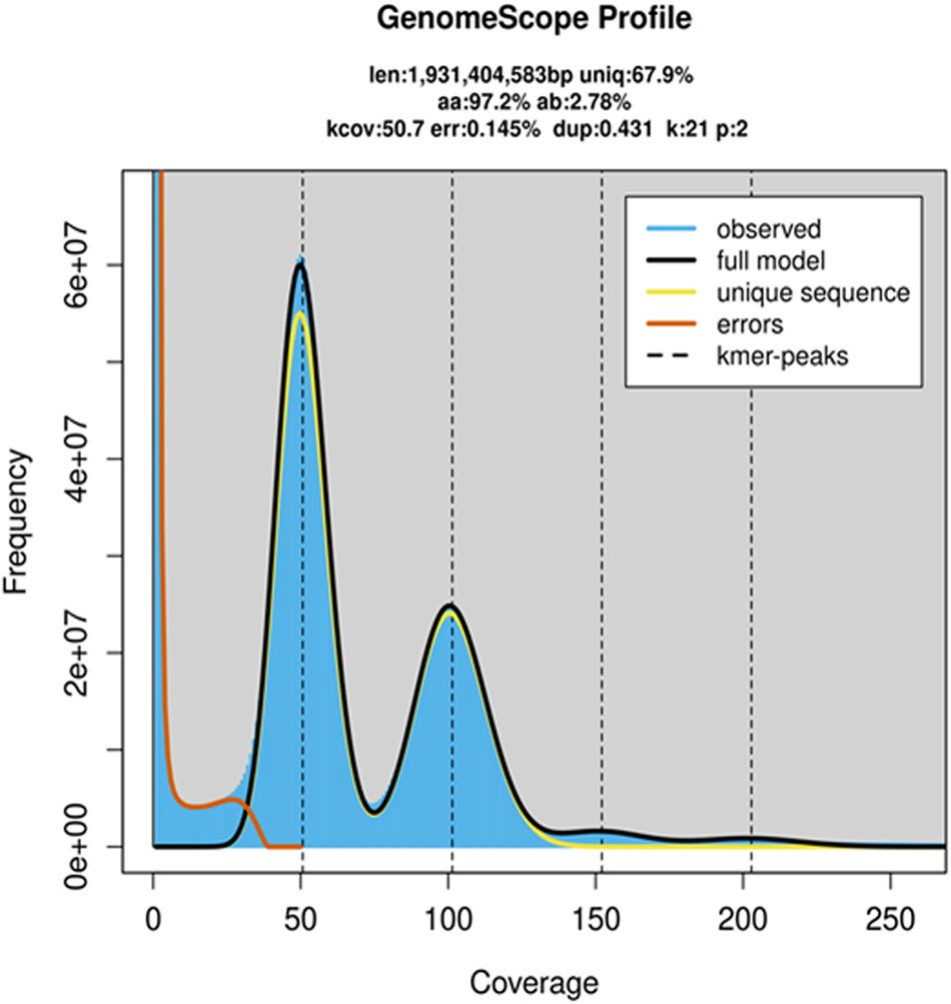
Genome size estimation of *Pardosa pseudoannulata* using Illumina reads.

**Fig. 2:**
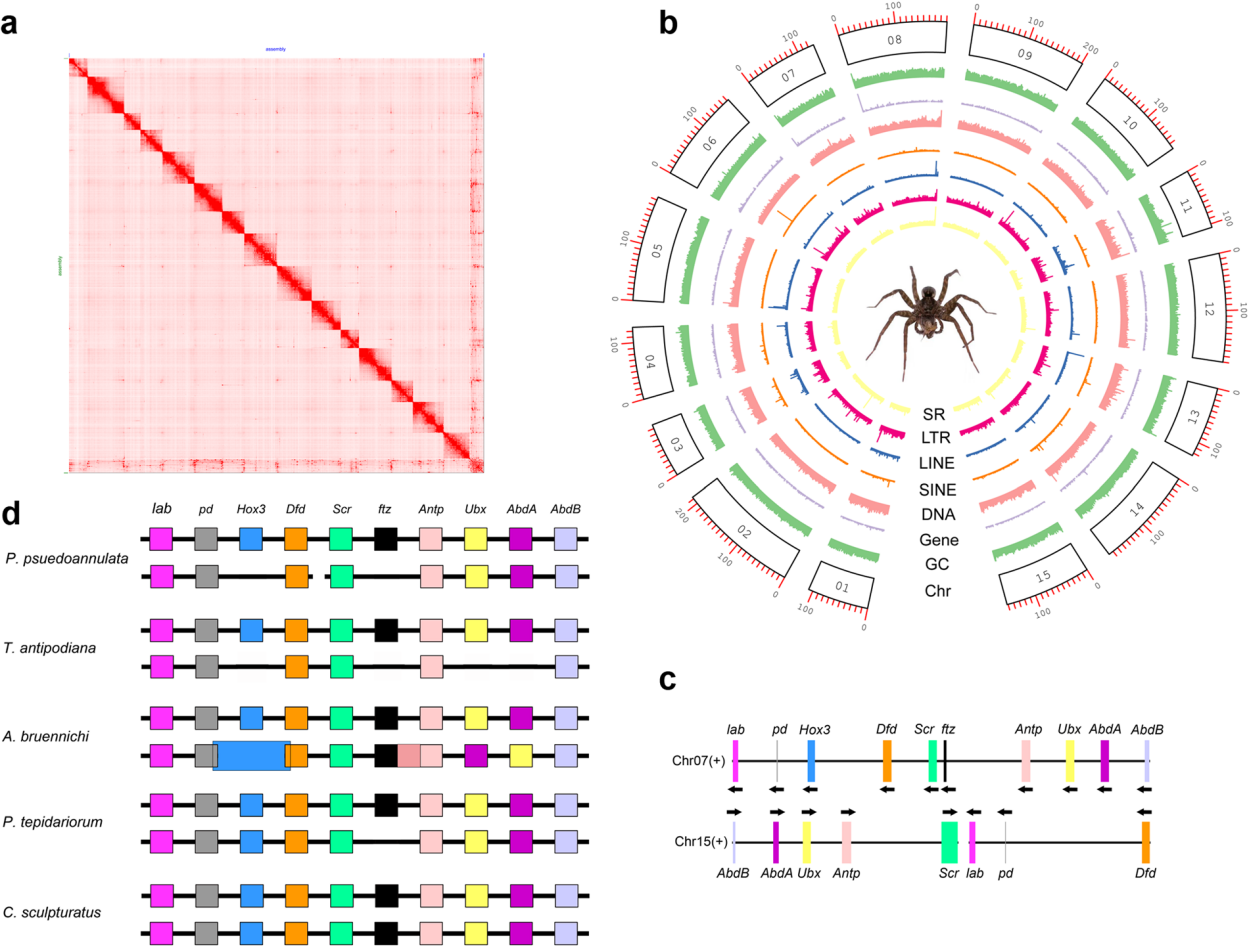
Characterization of *Pardosa pseudoannulata* genome. (**a**) Hi-C heatmap of the *P. pseudoannulata* genome. (**b**) Circos plot of the genomic characteristics. A female spider hunting planthoppers in the center, photographed by Dr. Lixin Huang. From the outer ring to the inner ring are the distributions of chromomsome length, GC content, gene density, TEs (DNA, SINE, LINE, LTR), AND simple repeats. (**c**) Hox genes in *P. pseudoannulata* genome. Black horizontal lines represent chromesomes and rectacular blocks represent Hox genes. Arrows indicate the direction of genes. (**d**) Hox genes in Arachnida species.

**Fig. 3:**
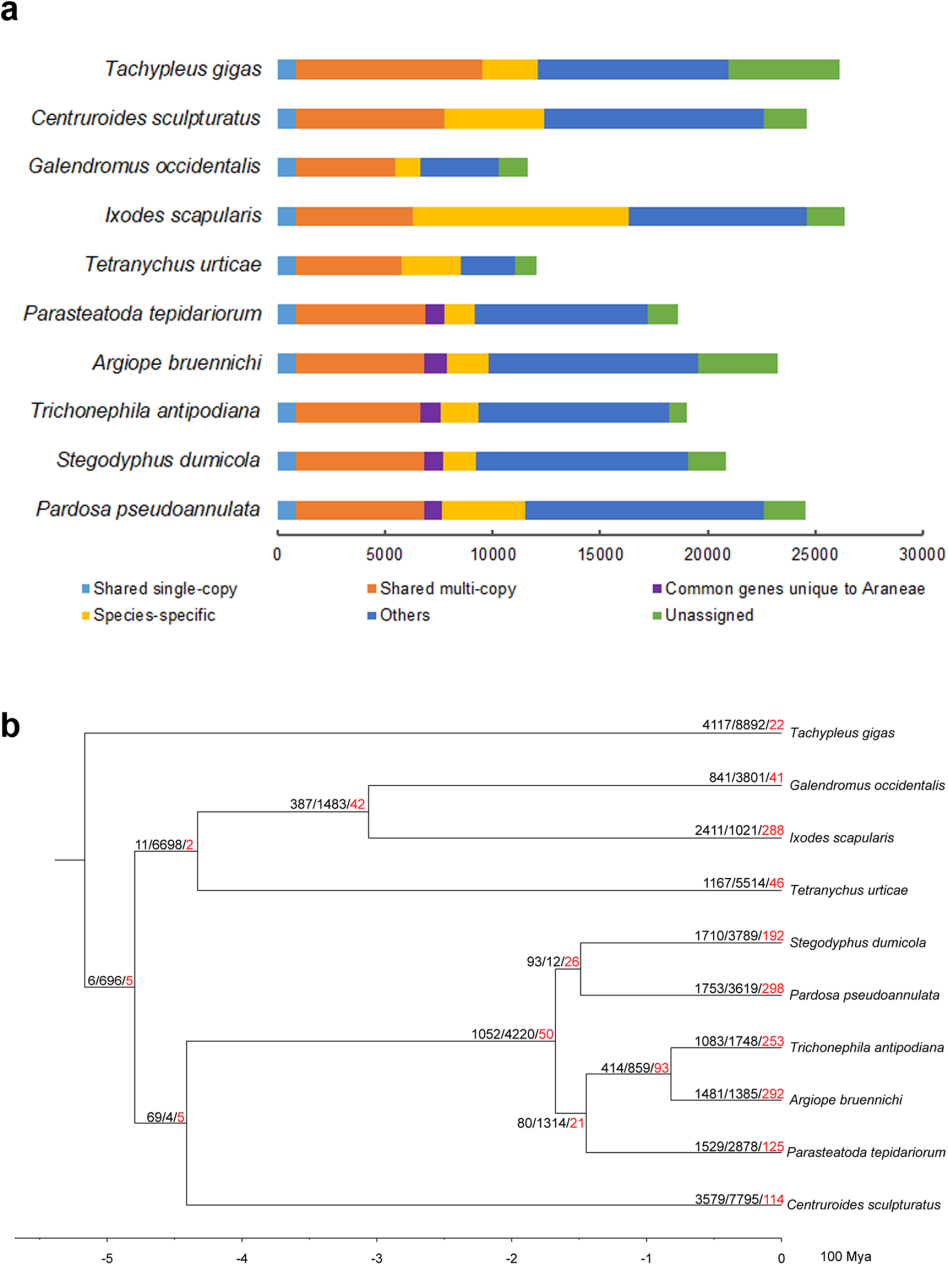
Phylogeny and gene family evolution of *Pardosa pseudoannulata*. (**a**) Comparison of orthologous genes between *P. pseudonannulata* and 9 other species. Horizontal coordinates represent the number of genes classified into 6 groups (single-copy, multi-copy, species-specific, unassigned, other, and common genes unique to Araneae). (**b**) Phylogenetic analysis of gene family. The estimated species divergence times (millions of years ago, MYA) are indicated under the tree. Node values indicate gene families showing expansion, contraction, and rapid evolution (red).

**Fig. 4:**
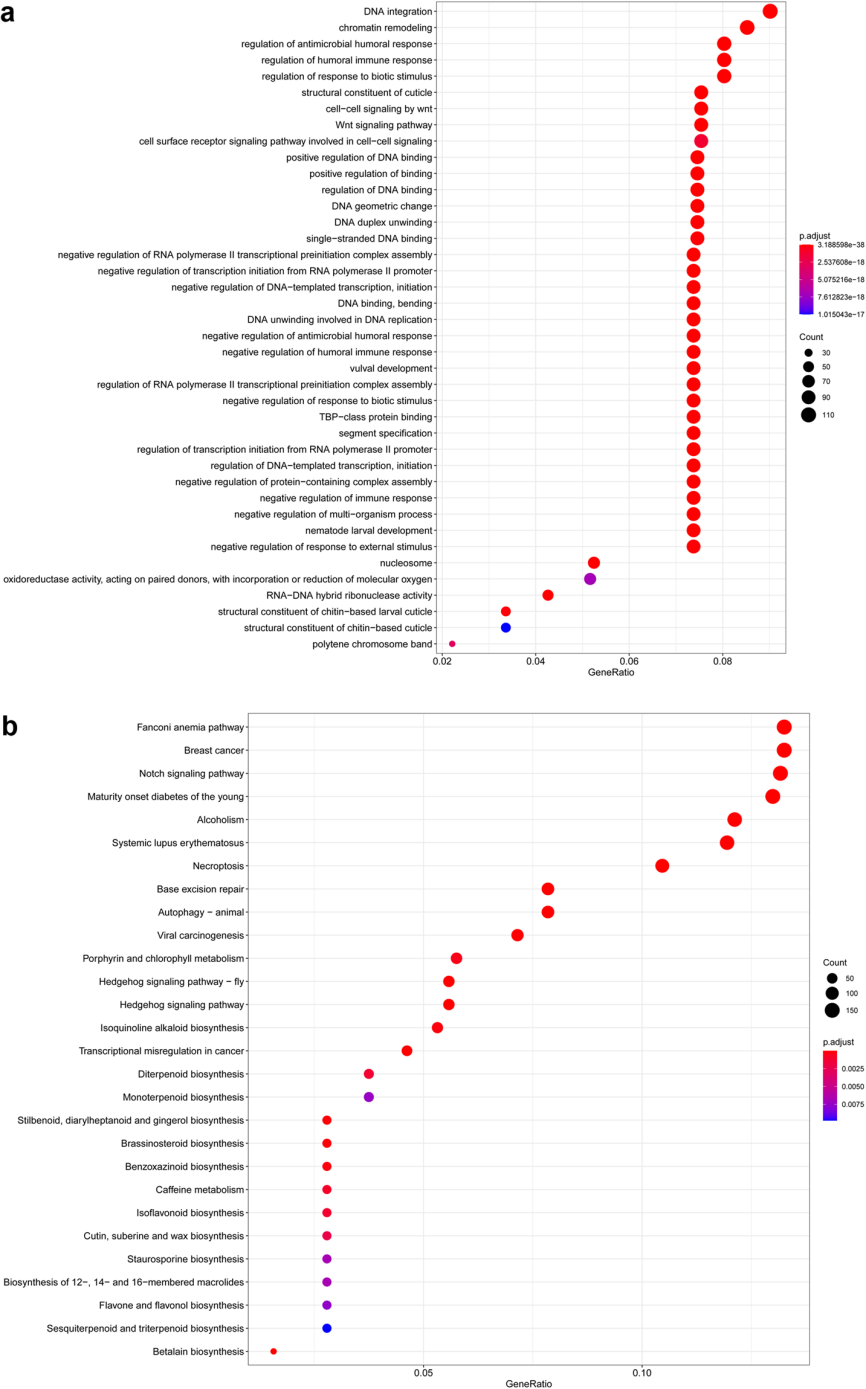
GO (**a**) and KEGG (**b**) annotation of expanded gene families in *P. pseudoannulata*.

**Fig. 5:**
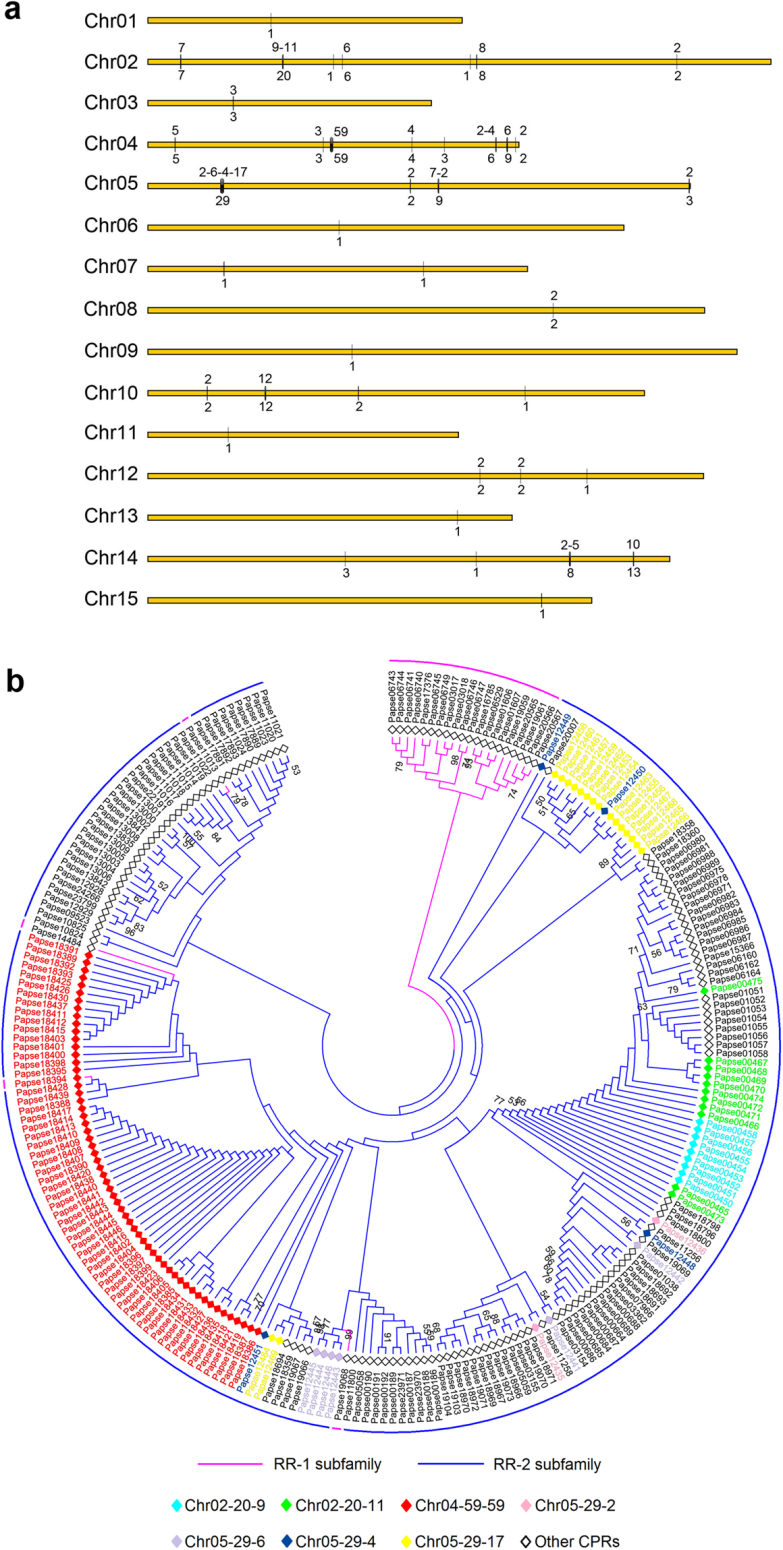
Expansion of genes encoding cuticle proteins with R&R consensus. (**a**) Schematic representation of the location of CPR genes on chromosomes. The light grey bars represent chromosomes; bars or lines represent the CPR gene clusters. The numbers below each bar or line represent the number of genes within the cluster, and the numbers above the bar or line represent the number of tandemly arrayed genes within the gene cluster, tandem arrays within one cluster was separated with “-”. (**b**) Phylogenetic analysis of CPR genes in a maximum-likelihood tree. The major genes clusters are indicated in colour.

**Fig. 6:**
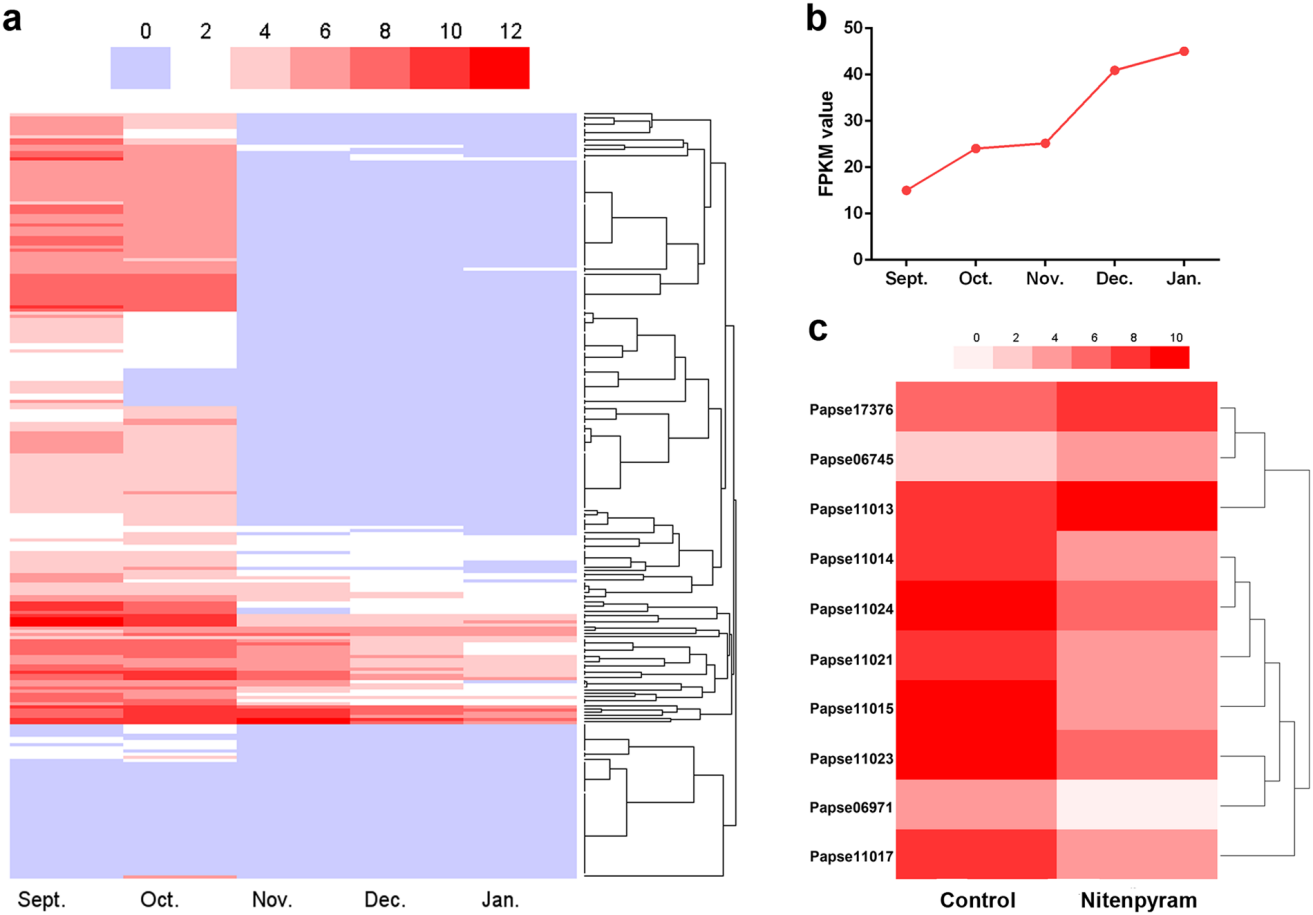
The expression pattern of CPR genes under environmental stresses. (**a**) The transcription levels of CPR genes in overwintering spiders. Spiders were collected in nature in five consecutive months. The transcriptional levels of genes were represented as log2(FPKM + 1). (**b**) The FPKM values of the gene Papse09523 in overwintering samples. (**c**) CPR genes exhibiting significant difference in transcription after nitenpyram exposure. The transcriptional levels of genes were represented as FPKM.

Through *k*-mer analysis, the estimated genome was 1.93 Gb and highly heterozygous (Fig. 1, Table 1). Genome sequencing yielded 1032.45 Gb of clean data from PacBio, Illumina, Hi-C, and Iso-seq data (Table 2). The final genome assembly was 2.42 Gb with 94.83% of the sequences assembled into 15 chromosomes (Fig. 2a,b, Table 4). The genome completeness assessed via BUSCO was 96.2%. The size of repeats was 1.28 Gb, accounting for 52.79% of the genome (Table 5). We predicted 24,530 protein-coding genes with the BUSCO completeness 95.8% (Tables 3, 7). The number of GO items and KEGG items was 13,925 and 19,197, respectively. Comparative genomic analysis revealed 265 gene families of significant expansion and 33 of significant contraction (Fig. 3b). Fifteen most significantly expanded gene families with the highest number of genes involved the gene regulation and protein processing including the cuticle protein gene family (Fig. 4, Table 10). Two clusters of Hox genes, one complete and one incomplete, were found in the genome. Cluster A was complete and occurred in a colinear order on chromosome 7 while Cluster B occurred on chromosome 15 with *ftz* and *Hox3* absent (Fig. 2c). We identified totally 243 genes encoding cuticle proteins containing the Rebers and Riddiford (RR) consensus, 24 categorized as RR-1 type and 219 as RR-2 type (Fig. 5). Except for four genes in scaffolds, CPR genes were distributed in all 15 chromosomes where many formed tandem arrays or gene clusters, the majority of which exhibited high sequence similarity. Using the transcriptomic data from overwintering spiders and nitenpyram-treated spiders, we investigated the transcriptional responses of CPR genes to the two stresses. The majority of CPR genes were expressed at higher level in Sept. and Oct., and along with the drop in temperature, a large number of CPR genes were down regulated and significantly to ground level in Nov. Dec. and Jan. (Fig. 6a). However, one gene *Papse09523* was upregulated in the course of overwintering though at a medium expression level among all CPRs. In the nitenpyram-exposure spiders, ten CPR genes were noticeable for their different transcriptional levels from those in control spiders (Fig. 6c). Three CPR genes were significantly up-regulated and they all encoded CPRs belonging to RR-1 subfamily. Seven CPR genes were noticeably down-regulated, six of which were located in a gene cluster of 12 genes in chromosome 10.

**Table 1:** Genome survey data.

Property	Min	Max
Homozygous (aa)	97.21%	97.23%
Heterozygous (ab)	2.77%	2.79%
Genome Haploid Length (bp)	1,929,276,367	1,931,404,583
Genome Repeat Length (bp)	619,259,159	619,942,274
Genome Unique Length (bp)	1,310,017,208	1,311,462,309
Model Fit	77.76%	96.64%
Read Error Rate	0.14%	0.14%

**Table 2:** Statistics of the DNA sequence data used for genome assembly.

Type	Number of reads	Data size (Gb)	Length per read (bp)	Sequencing coverage ( × )	Estimate genome size (Gb)
Survey	1,999,672,500	299.95	150	155	1.93
PacBio	19,508,376	456.40	23,394.92	236	
Hi-C	1,821,551,266	273.23	150	142	
Iso-seq	1,279,637	2.87	2,244.31	/	
Total	3,842,011,779	1,032	/	/

**Table 3:** Genome assembly information.

Assembly	Total length (Mb)	Number of scaffolds	N50 length (Mb)	Longest scaffold (Mb)	GC (%)	BUSCO (n = 1,013) (%)			
						Complete	Duplicate	Fragmented	Missing
raven	2,657.74	4,845	1.195	8.682	28.84	95.5	9.1	3	1.5
NextPolish	2,650.55	4,845	1.193	8.667	28.97	96.7	10	2.3	1
Purge_dups	2,421.46	3,786	1.35	8.667	28.79	95.9	5.5	3	1.1
3D-DNA	2,422.40	3,256	170.02	213.251	28.79	96.2	4.6	2.6	1.2
Final	2,416.64	1,958	169.991	213.218	28.79	96.2	4.6	2.6	1.2

**Table 4:** Summary statistics of the *P. pseudoannulata* genome assembly and annotation.

Characteristics	*Pardosa pseudoannulata*	*Argiope bruennichi*	*Dysdera silvatica*
**Genome assembly**			
Assembly size (Gb)	2.42	1.67	1.37
Number of scaffolds	1,958	2,231	15,360
Longest scaffold (Mb)	213.218	143.17	317.9
Scaffold N50 (Mb)	169.991	124.24	174.2
GC (%)	28.79	29.3	34.75
Gaps (%)	0.021	—	—
Number of chromosomes	15	13	7
Sequence in chromosomes (%)	93.39	98.4	87
BUSCO completeness (%)	96.2	91.1	92.9
**Gene annotation**			
Protein-coding genes	24,530	23,270	33,275
Mean protein length (aa)	408	—	—
Mean gene length (bp)	19,162.30	—	—
Exons/introns per gene	7.0/5.9	—	—
Exon (%)	1.83	—	—
Mean exon length (bp)	255.2	200	—
Intron (%)	17.62	—	—
Mean intron length (bp)	2,934	4,035	—
BUSCO completeness (%)	95.8	89.3	90.0
**Reference**	this study	Ref. ^15^	Ref. ^10^

-, information not available from the report.

**Table 5:** Repeat annotation in the P*ardosa pseudoannulata* genome.

Class	Copies	Length (bp)	Percentage of the genome
**DNA**	1,562,104	326,617,773	13.52%
**LINE**	237,580	52,924,679	2.19%
**LTR**	217,888	57,350,797	2.37%
**RC**	89,222	21,288,708	0.88%
**Retroposon**	65	7,607	0.00%
**SINE**	128,970	19,605,845	0.81%
**Unknown**	2,580,734	604,255,865	25.00%
**Unspecified**	4	230	0.00%
**Low_complexity**	105,373	5,470,380	0.23%
**Satellite**	21,210	1,863,302	0.08%
**Simple_repeat**	974,612	167,912,106	6.95%
**rRNA**	3,478	1,772,449	0.07%
**srpRNA**	166,365	16,233,470	0.67%
**Total**	6,087,607	1,275,303,283	52.77%

**Table 6:** Annotations of non-coding RNAs in *P. pseudoannulata*.

Class	Copies
rRNA	11
miRNA	6
snRNA	137
tRNA	5,821
Others	36
Total	6,011

**Table 7:** Statistics of protein-coding gene annotation.

Gene	Number	Percent (%)
Total	24,530	—
genes with InterProScan annotations	18,021	73.47
genes with GO items from InterProScan annotations	10,468	42.67
genes with KEGG pathway items from InterProScan annotations	10,969	44.72
genes with MetaCyc items from InterProScan annotations	11,315	46.13
genes with Reactome items from InterProScan annotations	14,380	58.62
genes matching Uniprot records	22,345	91.09
genes labelled as “Uncharacterized protein”	3,480	14.19
genes labelled as “unknown function”	2,410	9.82
genes with eggNOG annotations	18,485	75.36
genes with GO items from eggNOG annotations	9,779	39.87
genes with Enzyme Codes (EC) from eggNOG annotations	3,417	13.93
genes with KEGG ko terms from eggNOG annotations	10,459	42.64
genes with KEGG pathway terms from eggNOG annotations	6,516	26.56
genes with COG Functional Categories from eggNOG annotations	17,398	70.93
genes with GO items (combining InterProScan and eggNOG results)	13,925	56.77
genes with KEGG pathways items (combining InterProScan and eggNOG results)	19,197	78.26

**Table 8:** Statistics of gene family in *P. pseudoannulata*.

Parameters	Counts
Number of species	10
Number of genes	207,026
Number of genes in orthogroups	186,060
Number of unassigned genes	20,966
Percentage of genes in orthogroups	89.90%
Number of orthogroups	18,287
Number of species-specific orthogroups	5,155
Number of genes in species-specific orthogroups	31,731
Percentage of genes in species-specific orthogroups	15.30%
Mean orthogroup size	10.2
Number of orthogroups with all species present (single-copy and multiple-copy)	4,233
Number of single-copy orthogroups	805

**Table 9:** Statistics of orthologous gene families.

Species	Orders	Family	NCBI accession number	Shared single-copy	Shared multi-copy	Common genes unique to Araneae	Species-specific	Others	Unassigned
*Pardosa pseudoannulata*	Araneae	Lycosidae	JAGEOH000000000	805	5,991	834	3,873	11,088	1,939
*Stegodyphus dumicola*	Araneae	Eresidae	GCA_010614865.1	805	6,009	852	1,574	9,862	1,735
*Trichonephila antipodiana*	Araneae	Araneidae	PRJNA627506	805	5,822	922	1,817	8,836	799
*Argiope bruennichi*	Araneae	Araneidae	GCA_015342795.1	805	5,983	1,095	1,942	9,716	3,718
*Parasteatoda tepidariorum*	Araneae	Theridiidae	GCA_000365465.3	805	6,038	923	1,401	8,032	1,403
*Tetranychus urticae*	Trombidiformes	Tetranychidae	GCA_000239435.1	805	4,955	0	2,736	2,523	1,013
*Ixodes scapularis*	Ixodida	Ixodidae	GCA_016920785.2	805	5,493	0	10,047	8,253	1,782
*Galendromus occidentalis*	Mesostigmata	Phytoseiidae	GCA_000255335.2	805	4,685	0	1,160	3,609	1,378
*Centruroides sculpturatus*	Scorpiones	Buthidae	GCA_000671375.2	805	6,942	0	4,620	10,220	2,004
*Tachypleus gigas*	Xiphosurida	Limulidae	GCA_014155125.1	805	8,706	0	2,561	8,890	5,195

**Table 10:** Gene family expanson in *Pardosa pseudoannulata*.

Gene family	Number of genes
gag-polypeptide of LTR copia-type	292
HTH domain in Mos1 transposase	209
Zinc finger	203
Aspartyl protease	197
Putative peptidase	164
Reverse transcriptase (RNA-dependent DNA polymerase)	142
Endonuclease-reverse transcriptase	136
Core histone H2A/H2B/H3/H4	135
PIF1-like helicase	129
BTB/POZ domain	121
DDE superfamily endonuclease	115
Insect cuticle protein	93
Ribonuclease H	92
Cytochrome P450	59
Retroviral aspartyl protease	58

In summary, we assembled and annotated a chromosome-level genome of *P. pseudoannulata* here. The expanded cuticle protein gene family is highly related to *P. pseudoannulata*’s adaptation to varied environmental stresses as a wandering species. The duplicated clusters of Hox genes support the whole genome duplication event during the evolution of spiders. The genome data provide a reliable resource for functional and comparative studies in Arachnida.

## Methods

### Sample collection and sequencing

Five adult *Pardosa pseudoannulata* females (NCBI: txid330961) were collected in a paddy field in Jurong City, Jiangsu Province, China in 2020. Legs and muscle attached with cuticle from prosoma were collected and stored at −80°C. Genomic DNA was extracted with QIAGEN DNeasy Blood & Tissue kit. Two short paired-end insert libraries of 350-bp sequences were constructed for survey analysis using a Truseq DNA PCR-free kit and sequenced using the Illumina NovaSeq. 6000 platform. PacBio sequencing was performed with libraries constructed with insert size of 40 kb using a SMRTbell™ Template Prep Kit 2.0 on PacBio Sequel II. In addition, Hi-C sequencing was carried out with muscle from a single female. Muscle tissues were fixed with formaldehyde and lysed. The cross-linked DNA was digested with restriction enzyme MboI, biotinylated and proximity-ligated to form chimeric junctions that were physically sheared into size of 350 bp. Chimeric fragments representing the original cross-linked long-distance physical interactions were then processed into paired-end sequencing libraries, and 150-bp paired-end reads were generated using the Illumina NovaSeq. 6000.

RNA-seq samples were prepared in several individual batches of spiders. First, the Iso-seq sample was prepared by pooling the total RNA from spiders of different developmental stages (egg, juvenile, male and female adults) at equal molar ratio. Second, overwintering samples included spiderings (cephalothoax length 0.30–0.35 cm) collected monthly from paddy fields in Jurong city, Jiangsu Province, China from September 2019 to January 2020 when the average temperature went down from 26.5 to 3.0 degrees Celsius. Three replicate samples were prepared from each month with five individuals per replicated sample. Third, nitenpyram-exposure samples included spiderlings (14–20 days post egg-sac) fed with 1% acetone (solvent control) or 27.9 mg/L nitenpyram (treatment, sublethal dose) for 14 days. Three replicate samples were prepared for each control or treatment. Total RNA was extracted with TRIzol™ Reagent. RNA libraries were constructed with TruSeq RNA v2 kit and sequenced using the PacBio Sequel II platform. PacBio sequencing and Hi-C sequencing, and RNA sequencing, were performed by Berry Genomics (Beijing, China) and BGI Genomics (Shenzhen China).

We obtained 456.40 Gb PacBio DNA reads, 299.95 Gb Illumina DNA reads, 273.23 Gb Hi-C, and 2.87 Gb Iso-Seq data for genome assembly (Table 2).

### Genome survey and assembly

The Illumina reads were quality controlled using BBTools suite v38.67^20^. Duplicates were removed with “clumpify.sh”. Low-quality reads were discarded with “bbduk.sh” (qtrim = rl trimq = 20 minlen = 15 ecco = t maxns = 5 trimpolya = 10 trimpolyg = 10 trimpolyc = 10). The filtered reads were applied to estimating genome size. The *k*-mer distribution was estimated with khist.sh (BBTools). A 21-mer was selected for *k*-mer anlaysis with the maximum *k*-mer coverage cutoff set to 10,000 using Genomescope v2.0^21^. The estimated genome was 1.93 Gb and highly heterozygous (Fig. 1). The size of repeat sequence was estimated to be 619.26 Mb, accounting for 32.10% of the estimated genome (Table 1).

PacBio sequencing reads were first corrected with NextDenovo v2.3.1 (https://github.com/Nextomics/NextDenovo) and then subjected to assembly in Raven v1.3.0 (–weaken -p 0)^22^. Two rounds of polishing were carried out with Illumina short reads using NextPolish v1.3.0^23^ to improve the accuracy of the assembly. The heterozygous regions of the assembly were removed with two rounds of Purge_dups v1.2.5 (-a 70)^24^.

The chromosome-level assembly of the genome was generated by incorporating the Hi-C sequences. The Hi-C reads were firstly quality-controlled to remove duplicates and then aligned to the assembly using Jucier v1.6.2^25^. Chromosome-level pseudo-chromosomes were assembled with the defaults using 3D-DNA v180922^26^. Resulting assembly was manually corrected using Juicebox v1.11.08^25^ and completed using 3D-DNA. The final genome assembly was 2.42 Gb with 94.83% of the sequences assembled into 15 chromosomes (Fig. 2a,Tables 3, 4).

### Genome annotation

A *de novo* repeat database was initially constructed by using RepeatModeler v2.0.2a^27^ with LTR searching activated (-LTRStruct). The *de novo* repeat database was then combined with RepBase-20181026^28^ to obtain the reference repeat database. The repetitive elements were predicted and masked by searching against the reference repeat database using RepeatMasker v4.1.0^29^. The size of repeats was 1.28 Gb, accounting for 52.79% of the genome (Table 5). The most abundant repeat types were unknown (25.01%), DNA elements (13.52%), simple repeats (6.95%), LTR elements (2.37%), and LINEs (2.16%).

The non-coding RNAs (ncRNAs) were identified by predicting rRNA, snRNA and miRNA against Rfam database using infernal v1.1.4^30^ and annotating tRNA using tRNAscan-SE v2.0.9^31^ in the genome. tRNAs of low confidence were removed using the “EukHighConfidenceFilter” script in tRNAscan-SE. In total, 6,011 ncRNAs were predicted in the genome (Table 6).

Protein-coding genes were predicted via a strategy integrating three methods (*ab initio*, transcript-based, and protein homology-based evidence) with the repeat-masked genome in MAKER v3.01.03^32^. *Ab initio* prediction was carried out by Augustus v3.4.0^33^ and GeneMark-ES/ET/EP 4.68_lic^34^, both gene finders trained in BRAKER v2.1.5^35^ with the mapped RNA-seq and protein orthologs via ProtHint v2.4.0. RNA-seq data were firstly mapped to the genome assembly using HISAT2 v2.2.1^36^. Using the genome assembly as a reference, the mapped RNA-seq data were assembled into transcripts via StringTie v2.1.6^37^. Lastly, the reference protein sequences from *Stegodyphus dumicola* (GCA_010614865.1), *Parasteatoda tepidariorum* (GCA_000365465.3), *Argiope bruennichi* (GCA_015342795.1), *Daphnia magna* (GCA_020631705.2), *Ixodes scapularis* (GCA_016920785.2), *Trichonephila antipodiana* (PRJNA627506) were downloaded from NCBI database and served as the protein homology-based evidence for MAKER. The structural annotation predicted 24,530 protein-coding genes. The average number of exon per gene is 6.1, with a mean exon length of 262.7 bp and mean intron length of 3,456.9 bp (Table 4).

Predicted proteins were firstly searched against protein databases including UniProtKB (SwissProt + TrEMBL) and NR with a very sensitive mode (–very-sensitive -e 1e-5) using Diamond v2.0.11.149^38^. 22,345 proteins (91.09%) matched the UniProtKB entries. The predicted proteins were then subjected to domain searching via InterProScan 5.48-83.0^39^ against the databases Pfam^40^, SMART^41^, Superfamily^42^, and Conserved Domain Database (CDD)^43^. Structural domains of 18,021 proteins were identified through InterProScan. In addition, the predicted proteins were analysed for Gene Ontology (GO) and KEGG pathway annotation via eggNOG-mapper v2.1.5^44^ in eggNOG v5.0^44^. The number of GO items and KEGG items was 13,925 and 19,197, respectively (Table 7).

### Phylogenetic analyses and GO/KEGG enrichment analyses

Orthologous gene families were obtained using OrthoFinder v2.3.8^45^ from the protein-coding genes from 10 representative species including five Araneae species (*A. bruennichi*, *P. tepidariorum*, *P. pseudoannulata*, *S. dumicola*, and *T. antipodiana*), one Xiphosura (*Tachypleus gigas*), one Scorpiones (*Centruroides sculpturatus*), and three Acari (*I. scapularis*, *Galendromus occidentalis*, and *Tetranychus urticae*) (Table 9).

The phylogenetic tree of these ten species were constructed with 805 single-cope genes. The genes were firstly aligned using the strategy L-INS-I in MAFFT v7.394^46^ and trimmed to remove the region of low-homology using ‘-m BLOSUM90 -h 0.4’ in BMGE v1.12^47^. The resulting alignments were then concatenated using FASconCAT-G v1.04^48^. The phylogenetic tree was constructed with the concatenated alignments as supermatrix using IQ-TREE v2.0.7^49^ after removing the genes failing the SRH (stationary, reversible, and homogeneous) model using ‘–symtest-remove-bad–symtest-pval 0.10’. Protein substitution model was set as LG, combined with the partitioning algorithm (-m MFP–mset LG–msub nuclear–rclusterf 10). Node support values were assessed using the ultrafast bootstrap and SH-aLRT (Shimodaira-Hasegawa-like approximate likelihood ratio test) algorithms.

The divergence time was estimated using MCMCTree MCMCTree (clock = 2, BDparas = 1 1 0.1, kappa_gamma = 6 2, alpha_gamma = 1 1, rgene_gamma = 2 20 1, sigma2_gamma = 1 10 1) in the package PAML v4.9j^50^. Fossil records were retrieved from the PaleobioDB (https://www.paleobiodb.org/navigator/) with Chelicerata (genus Paleomerus) 516.0–541.0 million years ago (mya), Arachnida (Acariformes, *Pseudoprotacarus scoticus*) 407.6-419.2 mya, and Parasitiformes (*Deinocroton draculi*) 93.5–145.5 mya.

The likelihood of gene family expansion and contraction was identified using CAFÉ v4.2.1^51^ on the bases of the single birth-death parameter λ and a p-value threshold of 0.01. GO and KEGG enrichment of the significantly expanded gene families was analyzed using clusterProfiler v3.10.1 in R^52^. Among the genomes of the ten selected species, 186,060 genes were classified into 18,287 gene families, including 805 single-copy gene families and 3,428 multi-copy gene families (Table 8). In *P. pseudoannulata*, 22,591 out of the 24,530 genes were grouped in 10,741 families with 3,873 species-specific genes in 484 gene families (Fig. 3a,Table 9). *P. pseudoannulata* exceeded the other spider species in terms of the number of species-specific gene families.

Phylogenetic tree was constructed with 734 single-copy genes with 330,721 amino acid sites after IQ-TREE removed 71 single-copy genes. The UFB/SH-aLRT ratios of the branches in the maximum likelihood tree were all 100/100 with the exception for that of *Centruroides sculpturatus* and *Stegodyphus dumicola*-*Pardosa pseudoannulata* being 98.8/94 and 99.5/98, respectively. The number of gene families experienced expansion and contraction was 1,753 and 3,619, respectively, with 265 gene families of significant expansion and 33 of significant contraction (Fig. 3b). Fifteen most significantly expanded gene families with the highest number of genes involved the gene regulation and protein processing (Table 10). Among them, the cuticle protein and cytochrome P450 genes underwent expansion in *P. pseudoannulata*, which is likely to be in consistent with its adaptation to environment stresses, as also the case in *T. antipodiana*^11^. In addition, GO/ KEGG enrichment analyses of the expanded gene families further underlined the importance of cuticle protein genes as we detected the “structural constituent of cuticle” in GO and the “cutin, suberine and wax biosynthesis” in KEGG pathway enrichment (Fig. 3).

### Annotation of Hox genes

According to the annotation method of Hox genes in *Argiope bruennichi*^15^, the most complete sequences of the ten arthropod Hox gene classes from spiders were chosen as the Hox gene set. A TBLASN search against the genome assembly was performed to retrieve the Hox genes in *P. pseudoannulata*. The genomic position of best hits (E-value < 1.00 × 10^−20^ and identity >60%) were compared with the AUGUSTUS gene predictions for those locations. Only the Hox gene with the longest match-length in the same genomic position was retained. In *P. psuedoannulata*, the Hox genes have been manually annotated, and their genomic positions were retrieved (Fig. 2c). Two clusters of Hox genes, one complete and one incomplete, were found in *P. psuedoannulata*, in consistence to the results from three other spiders. One of the two clusters (Cluster A) was complete and occurred in a colinear order on chromosome 7. The other cluster (Cluster B) occurred on chromosome 15 with *ftz* and *Hox3* absent. Genes in Cluster B was divided by non-Hox genes into two subculsters as *AbdB-AbdA-Ubx-Antp* and *lab-pd-Dfd* in a reversed order on chromosome 15. In contrast to the complete cluster of Hox genes shared in the four spider genomes, the incomplete cluster occurs in a species-specific manner with *ftz* absent in *P. tepidariorum*^53^, *Hox3*, *ftz*, *Ubx*, and *AbdA* absent in *T. antipodiana*^11^, and *AbdA* and *AbdB* switching position in *A. bruennichi*^15^ (Fig. 2d). The Hox cluster duplication in *P. pseudoannulata* supports the whole-genome duplication (WGD) predicted to have occurred in the common ancestor of spiders and scorpions^11^ as evidenced also in three spiders (*P. tepidariorum*, *A. bruennichi* and *T. antipodiana*) and one scorpion (*C. sculpturatus*).

### Annotation and characterization of cuticle protein genes

We further examined the genes involved in the significantly expanded gene family “Insect cuticle protein” and structural domain analysis suggested that most genes encoding cuticle proteins with Rebers and Riddiford (R&R) consensus (CPRs). Genes encoding CPRs were identified following the method previously described in Li *et al*.^19^ with the manual verification of the R&R consensus. The protein sequences of all CPRs were aligned using ClustalW in MEGA6 and a phylogenetic tree was constructed with maximum-likelihood method, the bootstrap value of 1000. FPKM (Fragments Per Kilobase of exon model per Million mapped fragments) values of all CPR genes were retrieved from the transcriptomic data obtained from overwintering samples (NCBI accession: PRJNA907545) and nitenpyram-exposure samples (NCBI accession: PRJNA1015725)^54^. The log_2_(FPKM value + 1) was subjected to draw the heatmaps illuminating the transcriptional change of CPR genes in overwintering spiderlings and nitenpyram-exposed spiderlings. CPR genes with FPKM values less than 1 were considered not expressed and were excluded from analyses.

We identified totally 243 CPR genes including all of the 152 genes reported previously^19^. Twenty-four CPRs were categorized as RR-1 type and 219 as RR-2 type. Except for four genes in scaffolds, the majority of the CPR genes were distributed in all 15 chromosomes where many formed tandem arrays or gene clusters (Fig. 5). The CPR genes were unevenly located in chromosomes, with chromosome 4 being the most CPR-abundant (91 CPR genes), followed by chromosome 2 (45), chromosome 5 (43), chromosome 14 (25), and chromosome 10 (17), while one single CPR gene occurred in chromosomes 1, 6, 9, 11, 13, and 15 (Fig. 5a). The clustering of CPR genes in chromosomes may explain their uneven distribution, which has been reported in many arthropods^55–57^. In *P. pseudoannulata* chromosome 4, seven CPR gene clusters were present with the biggest cluster containing 59 genes in a tandem array. The most of CPRs clustered into two distinct branches, RR-1 subfamily and RR-2 subfamily, in the phylogenetic analysis (Fig. 5b). Four RR-1 members scattering in the RR-2 branch is probably attributed to their highly conserved flanking sequence with RR-2s. The huge number of closely located genes might be the result of gene duplication and the phylogenetic analysis supported it. The majority of the genes in tandem arrays or clusters exhibited high similarity, forming branches in the ML tree (Fig. 5b). For example, the 59 tandemly located gene in chromosome 4 closely clustered together in the ML tree. Nevertheless, some CPR genes were tandemly arrayed in chromosomes but their corresponding proteins scattered in the ML tree, such as the ones from cluster “Chr05-29-2” and “Chr05-29-4”.

As an essential component of cuticle, CPRs play important roles in the adaptation of arthropods to the habitat changes, especially to environmental stresses. *P. psuedoannulata* wanders in varied ecosystems including farm fields and faces complex environmental challenges, including low temperature in winter and insecticides exposure. Using the transcriptomic data from overwintering spiders and nitenpyram-treated spiders, we investigated the transcriptional responses of CPR genes to the two stresses. The majority of CPR genes were expressed at higher level in Sept. and Oct., and along with the drop in temperature, a large number of CPR genes were down regulated and significantly to ground level in Nov. Dec. and Jan. (Fig. 6a). The downregulation of CPR genes is in consistent with the observation that *P. pseudoannulata* did not molt during overwintering. However, one CPR gene *Papse09523* was upregulated in the course of overwintering though at a medium expression level among all CPRs (Fig. 6b), and the functions of this gene in cold tolerance triggers further investigation. Interestingly, *Papse16785* kept down-regulated during overwintering whereas it was significantly up-regulated after acute 0 °C exposure in our previous work (CPR12)^19^, suggesting that it might contribute to the different mechanisms underlying short-term and long-term coldness tolerance. In the nitenpyram-exposure spiders, ten CPR genes were noticeable for their different transcriptional levels from those in control spiders (Fig. 6c). Three CPR genes (*Papse06745*, *Papse11013* and *Papse17376*) were significantly up-regulated and they all encoded CPRs belonging to RR-1 subfamily. Seven CPR genes were noticeably down-regulated and six (*Papse11014*-*Papse11024*) were located in a gene cluster of 12 genes in chromosome 10. We previously reported several CPR genes were involved in *P. pseudoannulata*’s responses to different stresses^19^. Therefore, CPR genes might respond to chemical stresses in a coordinative way. The present study provides more valuable information on the genomic distribution and transcriptional responses to stresses of CPR genes for further functional studies.

## Data Records

The RNA-seq data were deposited in the SRA at NCBI for Iso-seq (SRR19534759)^58^, overwintering (SRR22498904-SRR22498907, SRR22498911, SRR22498913- SRR22498922)^59^, and nitenpyram-exposure (SRR26044769- SRR26044774)^60^. The genome assembly and annotation files are available in Figshare (10.6084/m9.figshare.24190083)^61^ and GenBank under the accession JAGEOH000000000^62^.

## Technical Validation

We detected possible contaminant sequences via blastn-like searches against the NCBI nucleotide (nt) and UniVec databases with a sequence identity of 0.8 (‘-min-seq-id 0.8’) using MMseqs. 2 v12-113e3^63^. Sequences with over 80% hits were checked via online BLASTN analysis in the NCBI nucleotide database. Sequences with over 90% hits in the databases were considered contaminants and removed from the assembled scaffolds.

The completeness of assembly was evaluated with BUSCO V5.4.4^64^ searching the arthropoda_odb 10 dataset (n = 1,013). The mapping rate was assessed by mapping the clean reads of Illumina and PacBio sequences to the genome assembly using Minimap2. The genome completeness assessed via BUSCO was 96.2% (Table 3).

On the basis of BUSCO analysis, the predicted protein-coding genes were identified 971 (95.8%) complete, 109 (10.8%) duplicated, 22 (2.2%) fragmented, and 20 (2.0%) missing orthologs (Table 4).

## Data Availability

All data processing commands and pipelines were carried out in accordance with the instructions and guidelines provided by the relevant bioinformatic softwares. There were no custom scripts or code utilized in this study.
